# Pre-eclampsia: Understanding clinical complexity

**DOI:** 10.1093/emph/eoy028

**Published:** 2018-09-12

**Authors:** Arjen R Buschman, Annelies Rep

**Affiliations:** 1 School of Medicine, Amsterdam University, Amsterdam, The Netherlands; 2 Department of Gynaecology, Noordwest Ziekenhuisgroep location Alkmaar, Alkmaar, The Netherlands

## PRE-ECLAMPSIA

Worldwide 2–8% of pregnancies are complicated by pre-eclampsia (PE), with an estimated maternal mortality of 50 000 cases annually [1]. PE is a part of the hypertensive syndrome of pregnancy and is defined as hypertension (>140/90 mmHg) with proteinuria (>300 mg/24 h). Symptoms of PE commence in the second half of pregnancy. These might encompass general symptoms of hypertension and/or proteinuria such as headache, exhaustion, nausea, vomiting, visual disorders and edema. Multiple organs can be affected causing severe complications such as eclampsia or progress to HELLP-syndrome.

**Figure 1.**
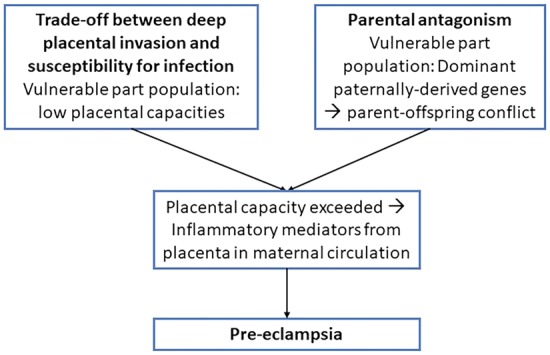
Model for development of PE. Evolutionary tradeoff between placental invasion and susceptibility for infection yields a vulnerable group at the low end of the spectrum of placental capacity. Superposed by dominant paternally-derived genes, placental capacity may be exceeded, causing PE

Delivery is the only definitive treatment. In preterm stages of pregnancy treatment of the disease occurs mainly symptomatically, aiming for analgesia and control of hypertension. In high-risk groups, a slight risk reduction of developing PE is achieved by administration of acetylsalycic acid in early pregnancy [2]. A century of research has not yet given physicians more powerful tools to protect both maternal and fetal conditions (Fig. 1).

## PATHOPHYSIOLOGY

For successful implantation of the placenta, maternal immune system has to develop local tolerance for the non-self-embryo [3]. Failure of this process prevents the extravillous trophoblast-cells from deeply invading the decidua, impairing maternal blood flow to the placenta. This can cause spontaneous abortion or development of a dysfunctional placenta. In the latter case, during second half of gestation, the fetal nutritional demands might outgrow placental capacity leading to local placental hypoxia. This triggers a release of inflammatory mediators from the placenta into maternal circulation, causing PE (Fig. 1).

## EVOLUTIONARY PERSPECTIVES

Down-regulation of local immunity in the decidua is likely to be shaped by an ‘evolutionary tradeoff’. Pregnancy yields higher susceptibility to infection and tolerance for fetal tissue as the trophoblast has shown to be disrupted by infection during pregnancy. Immunological tolerance is subject to regulation, reflected in the reduced risk to develop PE in a subsequent pregnancy from the same partner [3].

PE seems to occur in pregnancies with a reduced immunological tolerance. This yields a placenta with lower capacity, more vulnerable to a mismatch between fetal demands and maternal carrying capacity.

‘Parental antagonism’ hypothesizes that maternal and paternal interests are not entirely aligned in (partly) promiscuous species. The resulting conflict is (among other stages of the life cycle) fought using endocrinological influence of the trophoblast (fetal tissue) on maternal metabolism and circulation. By mechanisms like imprinting, genes are competing: paternally derived genes will manipulate maternal metabolism as to allocate more resources, while maternally derived genes aim to reduce expenses and risks of gestation and labor [4]. Both paternally and maternally derived genes benefit from a balanced conflict in which the maternal system can keep overall control, since this yields highest chances of healthy offspring. Incidental dominance of paternally derived genes can be considered a by-product of the evolutionary arms-race, eliciting a special case of ‘parent-offspring conflict’.

## FUTURE IMPLICATIONS

PE is likely to be too fundamentally rooted in evolutionary tradeoffs and parental antagonism to allow full prevention. Improvements in clinical practice could be achieved by a combination of first trimester screening and more causal treatment of the dysfunctional placenta. Early diagnostics will benefit from knowledge of the evolutionary background. Signs of dominant paternally derived genes could be found in (epi)genetics itself, but so far no candidate genes could be selected to reliably predict PE [5]. Screening of first trimester endocrine constitution of the maternal circulation for traces of unbalanced parental antagonism is showing more promising progress [6]. Few new therapies are being developed, but we are curiously awaiting the results of the EVERREST-trial in which the effects of genetherapy of the placenta are investigated [7].

**Conflict of interest**: None declared.
